# ITRAQ-based quantitative proteomics reveals apolipoprotein A-I and transferrin as potential serum markers in CA19-9 negative pancreatic ductal adenocarcinoma

**DOI:** 10.1097/MD.0000000000004527

**Published:** 2016-08-07

**Authors:** Chao Lin, Wen-Chuan Wu, Guo-Chao Zhao, Dan-Song Wang, Wen-Hui Lou, Da-Yong Jin

**Affiliations:** Department of General Surgery, Zhongshan Hospital Fudan University, Shanghai, China.

**Keywords:** aPOA-I, CA19-9 negative PDAC, serum markers, tF

## Abstract

Currently the diagnosis of pancreatic ductal adenocarcinoma (PDAC) relies on CA19-9 and radiological means, whereas some patients do not have elevated levels of CA19-9 secondary to pancreatic cancer. The purpose of this study was to identify potential serum biomarkers for CA19-9 negative PDAC.

A total of 114 serum samples were collected from 3 groups: CA19-9 negative PDAC patients (n = 34), CA19-9 positive PDAC patients (n = 44), and healthy volunteers (n = 36), whereas the first 12 samples from each group were used for isobaric tags for relative and absolute quantitation (iTRAQ) analysis. Thereafter, candidate biomarkers were selected for validation by enzyme-linked immunosorbent assay (ELISA) with the rest specimens.

Using the iTRAQ approach, a total of 5 proteins were identified as significantly different between CA19-9 negative PDAC patients and healthy subjects according to our defined criteria. Apolipoprotein A-I (APOA-I) and transferrin (TF) were selected to validate the proteomic results by ELISA in a further 78 serum specimens. It revealed that TF significantly correlated with the degree of histological differentiation (*P* = 0.042), and univariate and multivariate analyses indicated that TF is an independent prognostic factor for survival (hazard ratio, 0.302; 95% confidence interval, 0.118–0.774; *P* = 0.013) of patients with PDAC after curative surgery.

ITRAQ-based quantitative proteomics revealed that APOA-I and TF may be potential CA19-9 negative PDAC serum markers.

## Abstract 2

Supplemental Digital Content is available in the text

## 1 Introduction

Pancreatic ductal adenocarcinoma (PDAC) is one of the most aggressive malignant tumors with a 5-year survival under 5%.^[1,2]^ In 2013, there were a total of 45,220 new cases and 38,460 deaths attributed to pancreatic cancer in the USA.^[3]^ With few symptoms appearing at the early stage of the disease, 65% to 70% will have advanced disease (stage III-IV) at initial presentation, which means patients has lost the best chance to undergo surgery. Advanced pancreatic cancer has a very poor prognosis, with a median survival of 2 to 6 months for stage IV disease and 6 to 11 months for stage III disease.^[4]^ If PDAC is diagnosed at an early stage (tumor < 1 cm and without lymph node metastases), the 5-year survival increases to 50%.^[5]^ This poor prognosis is attributable to late stage presentation, lack of effective treatments, early recurrence, and the absence of clinically useful biomarker, which can detect pancreatic cancer in its precursor form or earliest stages.^[6–8]^

CA19-9 is a tumor-associated antigen, initially identified in the sera of patients with gastric and colon malignancies. Currently, CA19-9 is the most important biomarker for the diagnosis, prognosis, and management of PDAC,^[9–16]^ whose sensitivity and specificity for pancreatic cancer both are ∼80%,^[17]^ but it is not a sensitive marker to detect PDAC in the early stage. It has been reported that CA19-9 reacts with the sialylated Lewis^a^ blood group antigen present in the glycoprotein serum fraction.^[18]^ However, ∼5% to 10% of the general population has the Lewis^a-b-^ phenotype, which means that they are unable to synthesize the CA19-9 antigen and will not have elevated levels secondary to pancreatic cancer.^[19]^ It is necessary to find a marker that not only shows a higher sensitivity and specificity than CA19-9 before PDAC progresses to advanced stage, but also is appropriate for CA19-9 negative patients.

## 2 Patients and methods

### 2.1 Patients and tissue samples

A total of 36 fresh serum specimens were obtained from 12 normal control and 24 PDAC patients who underwent initial pancreatic resection at the Zhongshan Hospital between June 2012 and January 2013. Among the 24 serum specimens of PDAC patients, 12 were CA19-9 negative and the others were CA19-9 positive. Fresh sera were stored at –80°C until use. CA19-9 measurements were carried out at the clinical laboratory of Zhongshan Hospital. The upper limit of normal range used for CA19-9 was 37 U/mL. In the present study, 24 PDAC patients and 12 normal controls were divided into 6 subgroups (CA19-9 negative group I, II; CA19-9 positive group III, IV; normal control group V, VI) for proteomic analysis. Each subgroup consisted of 6 patients. For the serum apolipoprotein-I (APOAI) and transferrin (TF) level study, we examined an additional 54 patients with PDAC who underwent initial surgical resection and 24 healthy volunteers between July 2013 and June 2014 at the Zhongshan Hospital by enzyme-linked immunosorbent assay (ELISA) (Supplementary Figures 1 and 2). The investigational protocol was approved by the local institutional review boards, and informed consents were obtained from all study participants.

### 2.2 Protein extraction and labeling with isobaric tags for relative and absolute quantitation (iTRAQ) reagents

The samples were reduced at 60°C for an hour. Next, 1 mL cysteine blocking reagent was added to each tube before the protein samples were precipitated with ice-cold acetone. Then 20 mL of dissolution buffer was added to dissolute the sample. After reduction and alkylation, each sample was digested with trypsin (w(trypsin): w(protein) = 1: 20) at 37°C overnight.

The samples were then labeled with iTRAQ reagents (Applied Biosystems) as follows: CA19-9 negative PDAC subgroup I/II, iTRAQ reagent 113/116; CA19-9 positive PDAC subgroup III/IV, iTRAQ reagent 114/119; and normal control subgroups V/VI were labeled with iTRAQ 115/121, respectively. A total of 6 different isobaric tags were applied on 6 pooled protein samples and each of the 6 labeled digests were mixed, respectively.

### 2.3 2D LC–MS/MS

Agilent multiple affinity removal LC column-Human 14 (MARS) was used to deplete the high-abundance protein. One hundred μg sample each pool were fractionated on a waters UPLC using a C 18 column (waters beh c18 2.1 × 50 mm, 1.7 μm). Peptides were eluted at a flow rate of 600 μL/min with a linear gradient of 5% to 35% solvent B (acetonitrile) over 10 minute, the solvent A is 20 mM ammonium formate with pH adjusted to 10. The absorbance at 214 nm was monitored, and a total of 17 fractions were collected.

The fraction was separated by nano-HPLC (Eksigent Technologies, USA) on the secondary RP analytical column (Eksigent, C18, 3 μm, 150 mm × 75 μm). Peptides were subsequently eluted using the following gradient conditions with phase B (98% ACN with 0.1% formic acid) from 5 to 45% B (5–100 minutes) and the total flow rate was maintained at 300 nL/min. Electrospray voltage of 2.5 kV versus the inlet of the mass spectrometer was used. Triple TOF 4600 mass spectrometer was operated in information-dependent data acquisition mode to switch automatically between MS and MS/MS acquisition. MS spectra were acquired across the mass range of 350 to 1250 *m*/*z* using 250 ms accumulation time per spectrum. Tandem mass spectral scanned from 100 to 1250 *m*/*z* in high sensitivity mode with rolling collision energy. The 25 most intense precursors were selected for fragmentation per cycle with dynamic exclusion time of 25 seconds. We conducted the experiment in technical replicates.

### 2.4 ELISA analysis

ELISA was conducted in some marker candidates using human APOA-I ELISA kit (Assay Pro) and TF ELISA kit (Alpco). Their optical density was measured at 450 nm using a microplate reader (iMark Microplate Reader S/N 10288).

### 2.5 iTRAQ data analysis and statistical analysis

Protein identification and quantification of the iTRAQ data were performed using the ProteinPilot software version 4.2 (revision number: 1340; Applied Biosystems, USA). The Paragon algorithm (4.2.0.0, 1304) in the ProteinPilot software was used for peptide identification and isoform-specific quantification. The identified proteins were grouped by the software to minimize redundancy. All peptides used for the calculation of protein ratios were unique to the given protein or proteins within the group, and peptides that were common to other isoforms or proteins of the same family were ignored. The protein confidence threshold cutoff was 1.3 (unused ProtScore), with at least one peptide with 95% confidence. The false discovery rate for protein identification was calculated by searching against a reverse-concatenated database.

Student *t* test and 1-way ANOVA test were used to determine significant differences between different groups. Overall survival was calculated from the first resection of the primary tumor to death. All time-to-event end points were computed by the Kaplan–Meier method. Potential prognostic factors were identified by univariate analysis using the log-rank test. Independent prognostic factors were evaluated using a Cox proportional hazards regression model and a stepwise selection procedure. A 2-sided *P* < 0.05 was considered significant. Statistical analyses were performed using the software Statistical Package for the Social Sciences (SPSS, Chicago, IL).

## 3 Results

### 3.1 Protein identification

A total of 406 proteins with confidence interval values of no <95% were identified (Unused ProtScore *>* 1.3) by the iTRAQ-based experiment. To identify the differentially expressed proteins in the serum of CA19-9 negative PDAC patients, protein profiles between 2 types of sera (CA19-9 negative PDAC group vs normal control group) were compared. Proteins that were significantly and simultaneously upregulated or downregulated (fold-change ≥2 or ≤0.5) in both pairwise comparisons (subgroup I/subgroup V; subgroup II/subgroup VI) were regarded as potential differentially expressed proteins in the CA19-9 negative PDAC serum. As a result, trypsin-2 was found to be upregulated and 4 proteins including GTP-binding protein 5, apolipoprotein A-1, serotransferrin, and zinc finger protein 112 homolog (Supplementary Figures 3 and 4) were found to be downregulated in the CA19-9 negative PDAC serum compared with normal control serum.

### 3.2 The ELISA analysis of serum concentrations of APOA-I and TF

**Figure 1:**
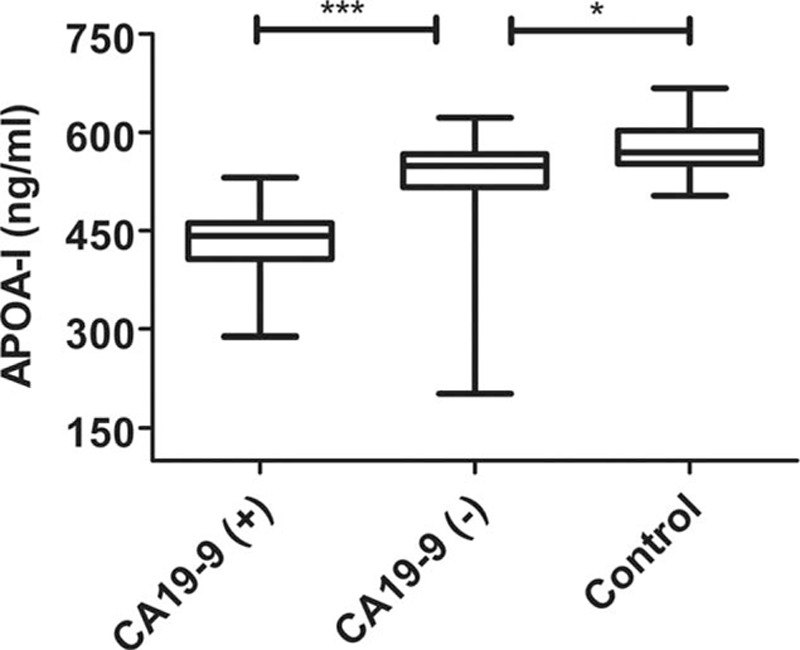
APOA-I levels were significant difference between CA19-9 negative PDAC patients, CA19-9 positive PDAC patients, and normal individuals.∗: *P* < 0.05; ∗∗∗:*P* < 0.001. APOA-I = apolipoprotein A-I, PDAC = pancreatic ductal adenocarcinoma.

**Figure 2:**
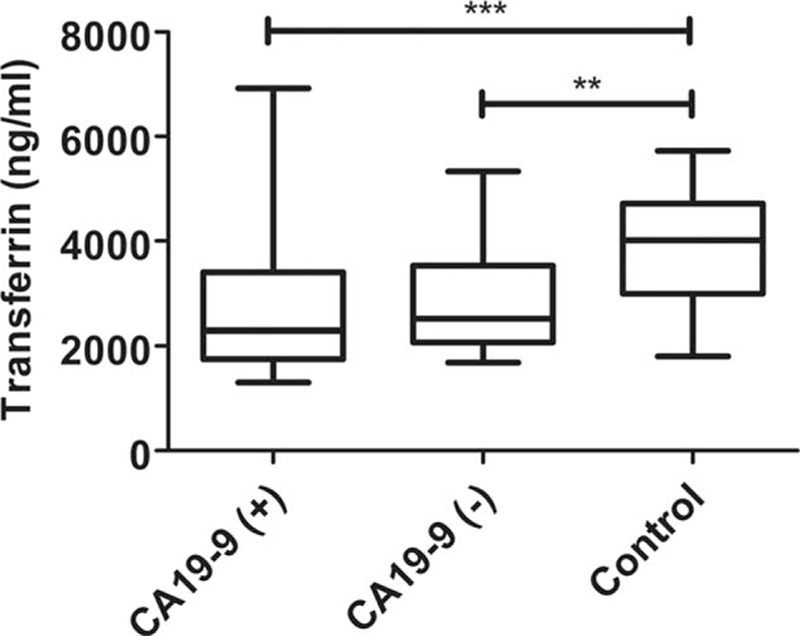
TF levels were significant difference between CA19-9 negative PDAC patients, CA19-9 positive PDAC patients, and normal individuals. ∗∗: *P* < 0.01; ∗∗∗:*P* < 0.001. PDAC = pancreatic ductal adenocarcinoma, TF = transferrin.

Among the identified proteins, APOA-I and TF were found to be associated to pancreatic cancer. However, whether they could be the biomarkers for CA19-9 negative PDAC has not been reported. For this reason, we directly assessed its level of expression by ELISA to validate our proteomic results. We used the entire 78 samples collected. A statistical significant difference between the CA19-9 positive PDAC group, CA19-9 negative PDAC group, and normal control group was seen in serum concentrations of APOA-I (435.1 ± 49.5 ng/mL in the CA19-9 positive PDAC group, 529.6 ± 84.0 ng/mL in the CA19-9 negative PDAC group, and 555.0 ± 87.1 ng/mL in the normal control group, (*P* < 0.001) and TF(3870.8 ± 1033.3 ng/mL in the CA19-9 positive PDAC group, 2920.8 ± 1097.6 ng/mL in the CA19-9 negative PDAC group, and 2769.5 ± 1329.9ng/mL in normal control, *P* = 0.002) (Figs. 1 and 2). The difference in APOA-I level between CA19-9 positive PDAC group and CA19-9 negative PDAC group also reached statistical significance (*P* < 0.001), suggesting the specificity of this marker for CA19-9 negative PDAC. The cutoff value and corresponding sensitivity and specificity values for both biomarkers were shown in Table 1

**Table 1:**
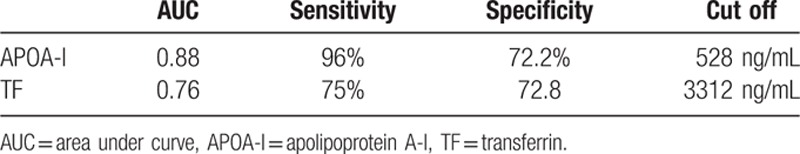
Diagnostic validity test of APOA-I and TF.

### 3.3 Clinical significances of APOA-I and TF level in PDAC

**Figure 3:**
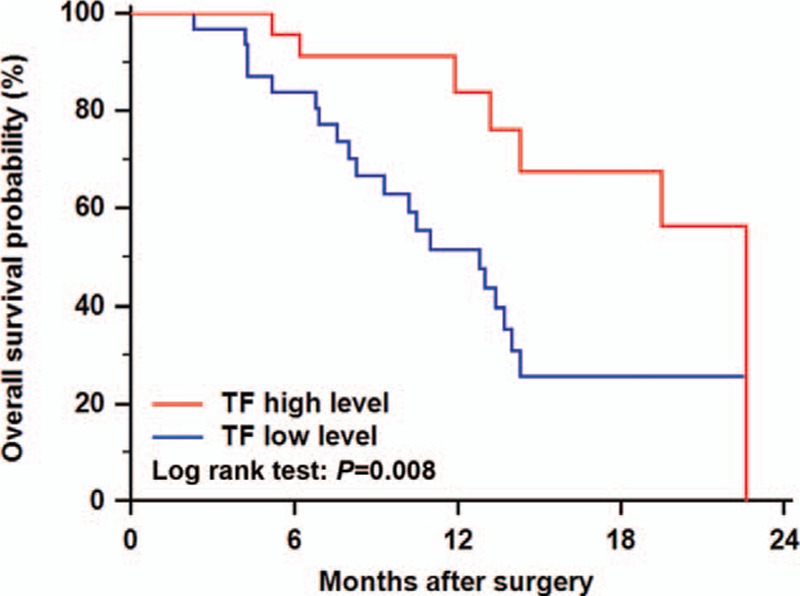
Correlation of TF level with clinical outcome of PDAC patients after curative resection. The patients with TF level over the mean level had better prognosis in terms of overall survival. PDAC = pancreatic ductal adenocarcinoma, TF = transferrin.

To evaluate the association of APOA-I and TF level with tumor biology, comparisons of the clinicopathological features with APOA-I and TF level were made. As shown in Table 2, differentiation was correlated to TF levels (*P* = 0.042). To estimate the prognostic value of APOA-I and TF, Kaplan–Meier survival analysis was performed. As shown in the Kaplan–Meier survival curve (Fig. 3), patients with higher level of TF were prone to better OS (*P* = 0.008). In addition, Cox multivariate regression analyses were performed to define independent risk related to overall survival. As shown in Table 3, TF (hazard ratio, 0.302; 95% confidence interval, 0.118–0.774; *P* = 0.013) and neural invasion (hazard ratio, 4.941; 95% confidence interval, 1.407–17.342; *P* = 0.013) were both identified as independent prognostic factors.

**Table 2:**
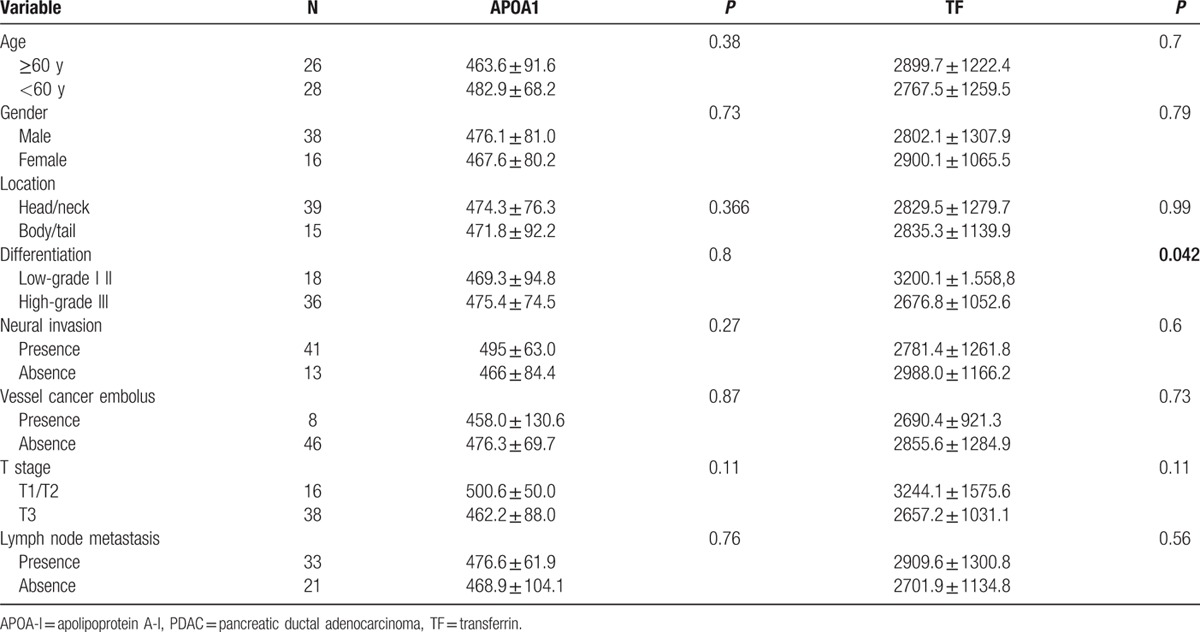
Clinical correlation between APOA-I, TF level, and clinicopathological parameters of PDAC patients (n = 54).

**Table 3:**
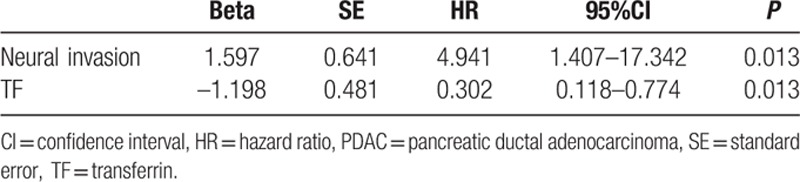
Multivariate analyses of prognostic factors for patients with PDAC (n = 54).

## 4 Discussion

To our certain knowledge, this is the first investigation that focused on differentially expressed serum protein in patients with CA19-9 negative PDAC. In the past 20 years, several proteomics technologies, such as 2D polyacrylamide gel electrophoresis and multidimensional liquid chromatography, have been used to research novel proteins with a potential diagnostic role in PDAC. The iTRAQ technique, which allows for the direct comparison of protein levels present in samples from control and diseased patients, is one of the newest of these high-sensitivity, high-accuracy techniques. It has the advantage of accurate identification and quantification of all the proteins expressed by a particular genome or the proteins in a complicated mixture. iTRAQ analysis has been widely used to identify potential biomarkers because of the high reproducibility and high sensitivity.^[20,21]^ However, it was a common phenomenon that the cut-off values were set at 20% to 50% average variance ^[22]^ to identify differentially expressed proteins, which resulted in a low specificity of the markers.^[23]^ As a result, we chose to set the cut-off value at 100% variance to screen potential serum biomarkers for CA19-9 negative PDAC patients. We divided the patients into 2 subgroups respectively in CA19-9 positive PDAC group, CA19-9 negative PDAC group and normal control group. Only when the proteins expressed over 2-fold differentiation between CA19-9 negative PDAC group and normal control in both subgroup comparisons could they become the candidates of the potential biomarkers. In this way, the differentially expressed proteins are more likely to be potential biomarkers for CA19-9 negative PDAC. However, the results of ELISA were not completely consistent with that of iTRAQ. On the one hand, the level of TF and APOA-I between CA19-9 negative PDAC and normal control group did not show 2-fold differentiation in the ELISA assay, on the other hand, the difference of TF and APOA-I between the CA19-9 negative PDAC group and the CA19-9 positive PDAC group were not significant in iTRAQ, whereas the APOA-I level was proved statistical between the 2 groups in the ELISA assay. There are 2 main reasons contributing to the phenomenon. First, the number of specimen for iTRAQ analyses was comparatively small, with the subgroups being made up of 6 serum specimens. When performing the iTRAQ analyses, we mixed the 6 specimens together as a group so that as long as any one of the specimens with extremely high or low level of some protein, the mean level would be affected greatly. Second, it was different methods themselves that leading to the difference. Different methods do not have the same efficiency in quantification of proteins.

APOA-I is secreted as a member of apolipoprotein A (APOA), a high-density lipoprotein apolipoprotein. Wu found APOA was significantly related to pancreatic adenocarcinoma.^[24]^ However, it is difficult to tease out the specific mechanism responsible for the link between APOA levels and PDAC risk, but their research suggested that dyslipidemias may play a role in pancreatic tract carcinogenesis through their inflammatory properties. Furthermore, low APOA-I levels were found to be associated with a higher risk of several cancers such as breast cancer, and gastic cancer.^[25,26]^ As a result, we infer that there are distinct etiological relationships between lipid profiles and pancreatic adenocarcinoma, in which APOA-I play an important part and it may become a potential biomarker for PDAC.

Transferrin is an iron-binding transport protein, which can bind 2 Fe^3+^ ions in association with the binding of an anion. Clerc's research found that it was one of the essential factors for the proliferation of pancreatic cancer cell lines.^[27]^ Furthermore, it was reported that TF was increased in the pancreatic juice of the patients with pancreatic cancer, as compared with apparently healthy adults.^[28]^ Based on findings of our study, we guess that TF is secreted to pancreatic juice rather than blood in patients with PDAC. The finding that TF expression correlated with differentiation of the tumor and the survival further confirmed the importance of this protein in association with PDAC.

In conclusion, our study clearly identified APOA-I and TF as the differentially expressed proteins in CA19-9 negative PDAC patients, which could be the potential biomarkers for CA19-9 negative PDAC serum markers. Furthermore, elevated expression of TF was strongly associated with a favorable outcome. Of course, our results provide the preliminary clue on the potential biomarkers of CA19-9 negative PDAC that needs to be validated in a larger cohort.

## Supplementary Material

Supplemental Digital Content
