# Supplementary material for: ITRAQ-based quantitative proteomics reveals apolipoprotein A-I and transferrin as potential serum markers in CA19-9 negative pancreatic ductal adenocarcinoma
Source: Medicine (Baltimore). 2016 Aug 7;95(31):e4527. doi: 10.1097/MD.0000000000004527 (PMC4979862; doi:10.1097/MD.0000000000004527)

Supplementary figure 1: Power is the probability of rejecting a false null hypothesis. N is the average group sample size. K is the number of groups. Total N is the total sample size of all groups combined. Alpha is the probability of rejecting a true null hypothesis. Beta is the probability of accepting a false null hypothesis. Sm is the standard deviation of the group means under alternative hypothesis. Standard deviation is the within group standard deviation. The effect size is the ration of Sm to standard deviation. Hypothesized means are the mean level of Transferrin in three subgroups according to the ELISA assay. The largest SD of the three subgroups, 1097, is selected to conduct the analysis. Even though we choose the smallest number of the three subgroups, 22, the power is 0.894.


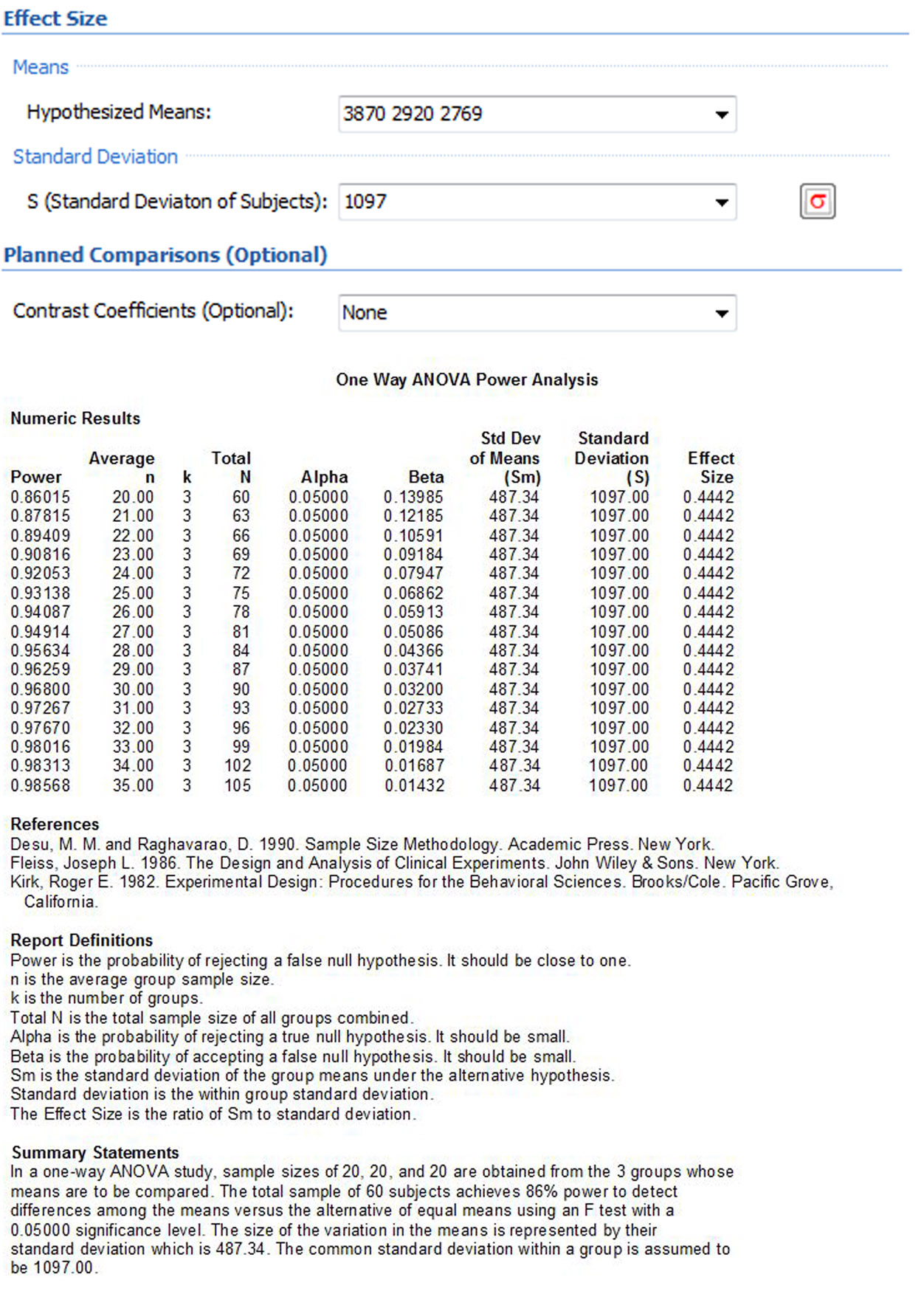


Supplementary figure 2: Power is the probability of rejecting a false null hypothesis. N is the average group sample size. K is the number of groups. Total N is the total sample size of all groups combined. Alpha is the probability of rejecting a true null hypothesis. Beta is the probability of accepting a false null hypothesis. Sm is the standard deviation of the group means under alternative hypothesis. Standard deviation is the within group standard deviation. The effect size is the ration of Sm to standard deviation. Hypothesized means are the mean level of APOA-I in three subgroups according to the ELISA assay. The largest SD of the three subgroups, 87, is selected to conduct the analysis. Even though we choose the smallest number of the three subgroups, 22, the power is 0.992.


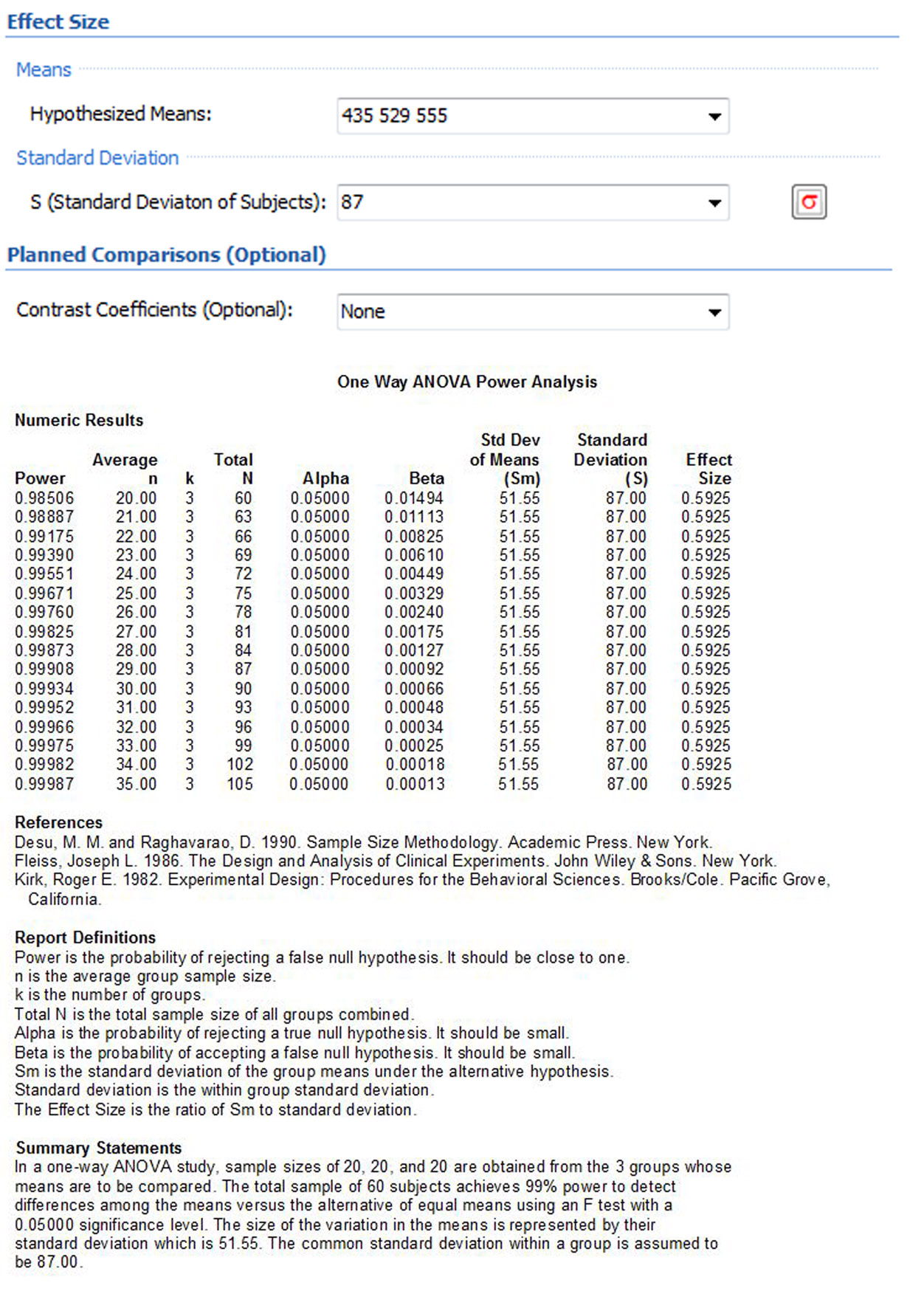


Supplementary figure 3: The representative spectra for APOA-I. CA19–9 negative PDAC subgroup I/II, iTRAQ reagent 113/116; CA19–9 positive PDAC subgroup III/IV, iTRAQ reagent 114/119; and normal control subgroups V/VI were labeled with iTRAQ 115/121 respectively.


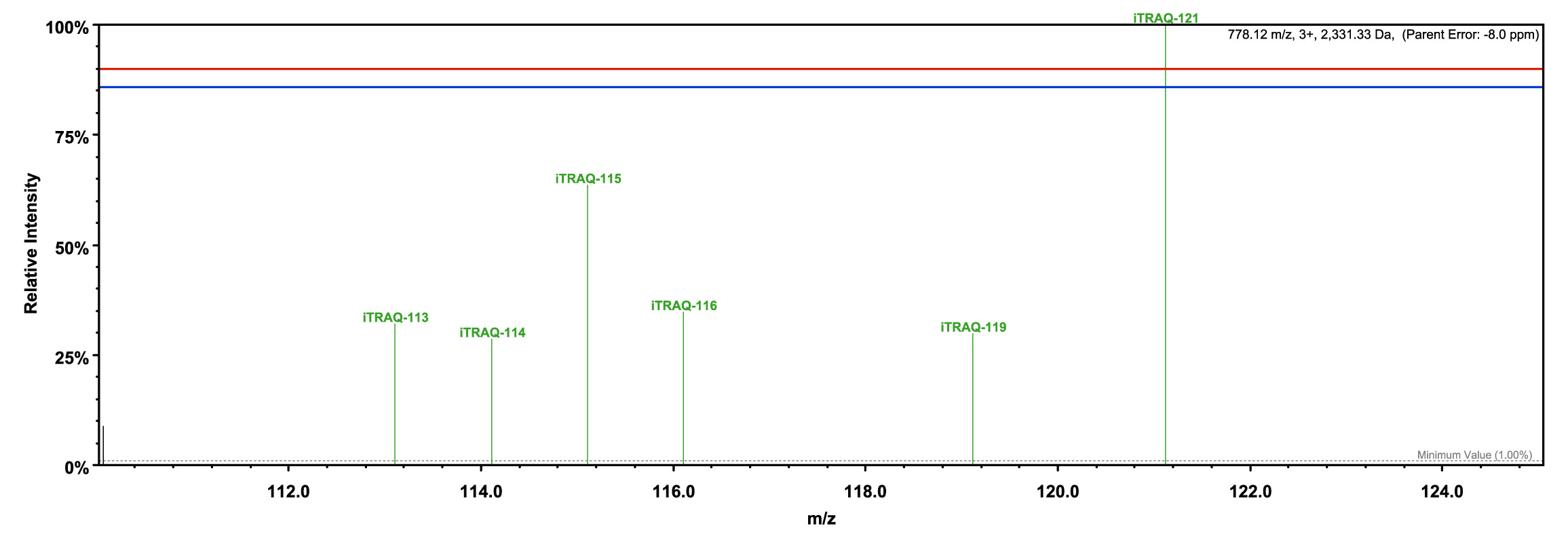


Supplementary figure 4: The representative spectra for TF. CA19–9 negative PDAC subgroup I/II, iTRAQ reagent 113/116; CA19–9 positive PDAC subgroup III/IV, iTRAQ reagent 114/119; and normal control subgroups V/VI were labeled with iTRAQ 115/121 respectively.


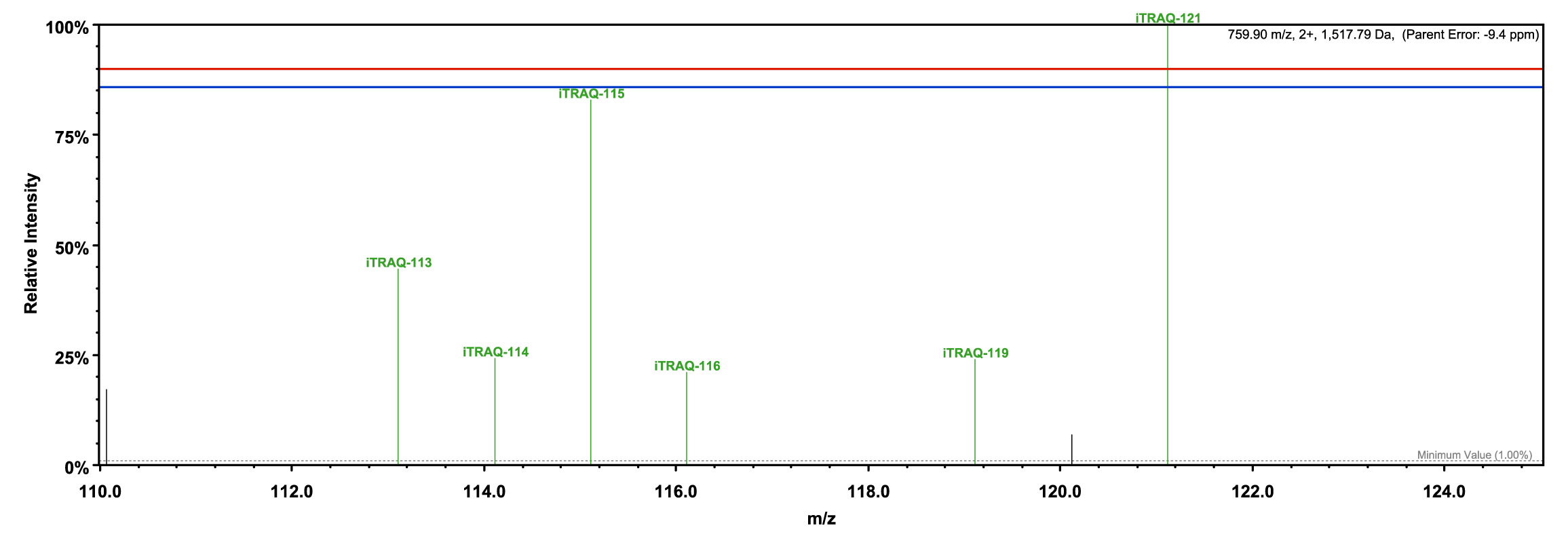

Supplement: Supplemental Digital Content [file medi-95-e4527-s001.doc]
